# Association Between Deviation of Fairness Perceptions from Group Average and Serious Psychological Distress in Japanese Worksites: a Cross-Sectional Study

**DOI:** 10.1007/s12529-019-09781-8

**Published:** 2019-03-14

**Authors:** Nobutada Yokouchi, Hideki Hashimoto

**Affiliations:** Department of Health and Social Behavior, Graduate School of Medicine, The University of Tokyo, 7-3-1 Hongo, Bunkyo-ku, Tokyo, 113-0033 Japan

**Keywords:** Fairness perception, Deviation, Worksite, Japan, Serious psychological distress

## Abstract

**Background:**

Workers with deviating fairness perceptions are likely to be excluded and become isolated at worksites, leading to psychological distress. The study aimed to examine the cross-sectional association between deviation of fairness perception from the group average and serious psychological distress in Japanese worksites.

**Methods:**

Secondary data analysis of an existing Japanese occupational cohort data using a multilevel logistic regression model was conducted for 8701 workers from 12 companies in Japan who participated in the baseline survey (from April 2010 to March 2012). Individual perception of interactional and procedural fairness was measured with the Japanese version of the Organizational Justice Scale. Group averages were calculated within workers’ reference groups, categorized by company affiliation, age group, gender, and occupational class. Psychological distress was measured using the K6 scale, and serious psychological distress was defined as a total K6 score of 13 or more.

**Results:**

Both low deviation and high deviation of interactional fairness perception were significantly and positively associated with serious psychological distress (odds ratio (OR) = 1.24, 95% confidence intervals (CI); 1.03–1.49 and OR = 1.57, 95% CI; 1.12–2.19), independently of individual-level fairness perception, group-level mean fairness perception, demographic characteristics, and health-related behaviors. Only high deviation of procedural fairness perception was significantly and positively related to serious psychological distress (OR = 1.51, 95% CI; 1.11–2.06).

**Conclusions:**

The results indicated that divergent perceptions of fairness at worksites may deserve further exploration for equal achievement of workers’ psychological well-being.

## Introduction

Organizational justice in the worksite has emerged as a psychosocial determinant of workers’ health that captures a relational and administrative dimension of the worksite environment, rather than the job-related psycho-socio-physical stressors that the demand–control and effort–reward imbalance models focus on [1–3]. Organizational justice is defined as workers’ perceptions of fairness in the worksite, and the concept is comprised of three domains: distributive fairness, procedural fairness, and interactional fairness [4]. Distributive fairness refers to the fairness of the outcomes that a worker receives [5]. Procedural fairness is concerned with the fairness of the decision-making procedures that lead to those outcomes [6] Interactional fairness focuses on the interpersonal treatment received from decision makers [4].

Because distributive fairness is thought to result from procedural fairness and interactional fairness [5], the latter two domains have been the main focus of occupational health research [7].

Accumulating evidence indicates the protective function of fairness perception against job stress and related ill health, as well as subsequent sickness absence [7–13]. Most studies have examined these associations at an individual level. However, recent studies have begun to investigate the multilevel effects of group-level fairness perception (i.e., justice climate) on aspects of individual worker health, such as somatic complaints, psychiatric morbidity, and work-related burnout [14–18].

In addition to the effects of fairness, researchers have considered the psychological process underlying workers’ pursuit of fairness. The instrumental model posits that workers seek fairness because it leads to desired outcomes, whereas relational model assumes that workers seek fairness because they value within-group relationships [19, 20]. The most recently proposed deontic model postulates that workers pursue fairness for its own sake, because they feel obligated to follow moral norms [19, 21, 22]. Moral norms refer to the individual moral obligations derived from socially shared norms [23, 24]. The deontic model has been suggested to have good affinity with workers at Japanese worksites, where workers’ behaviors are bounded primarily by the normative rules that are implicitly or explicitly imposed by the company [25].

According to the deontic model, workers evaluate whether events conform to or deviate from their moral norms [19, 23]. Hence, it is likely that workers with fairness perceptions that deviate from the group average possess moral norms that deviate from moral norms shared by the group. As these workers could threaten the cohesiveness and integrity of the group as a whole, regardless of whether the deviation is positive or negative [26, 27], they are likely to be excluded and become isolated within the group [28–32]. The exclusion and isolation can be prominent, especially in non-Western countries such as Japan, where collective social norms are prevalent [33]. This could eventually have detrimental effects on the health of workers with deviating perceptions [34]. However, to the best of our knowledge, no studies have examined the association between a worker’s relative position in the group and her/his mental health.

An examination of the effect of deviation is also crucial from a practical perspective. Conventional population-based interventions for worksite fairness focus mainly on the average level of fairness in the group [35]. Although this may generally have positive effects on workers’ health, workers with diverging perceptions may still be excluded and their isolation exacerbated. If so, inclusive treatment for individuals with divergent perceptions within a group may be needed to avoid social isolation by group norms in the worksite.

Therefore, the purpose of the present study was to examine the cross-sectional association between individual deviation of fairness perception from the group average and serious psychological distress in Japanese worksites. It was hypothesized that deviation of fairness perception is positively associated with serious psychological distress, independently of individual-level fairness perception, group-level mean fairness perception, demographic characteristics, and health-related behaviors.

## Methods

### Procedures

This was a cross-sectional study using secondary data from the baseline survey of an occupational cohort study on social class and health in Japan, the Japanese Study of Health, Occupations, and Psychosocial Factors Related Equity (J-HOPE) [36, 37]. J-HOPE was conducted in four waves from April 2010 to March 2014. The baseline survey was conducted from April 2010 to March 2012. The present study used the J-HOPE baseline dataset as of 22 August 2016 for the analysis. The J-HOPE study population comprised 14,189 full-time workers from 12 companies located in Japan. Of the study population, 10,742 workers responded to the baseline survey.

The current study was waived for ethical approval because it uses the anonymized secondary data of an ethically approved occupational cohort study.

### Measures

#### Deviation of Fairness Perception

Fairness perception was measured using the Japanese version of the Organizational Justice Scale [38]. The Japanese version of the Organizational Justice Scale is based on Elovainio et al.’s scale [39], which modified the original scale developed by Moorman (often referred to as the Organizational Justice Questionnaire: OJQ) [6]. The Japanese version of the OJQ contains a six-item interactional fairness scale and a seven-item procedural fairness scale. The interactional fairness scale assesses the fairness perceptions of the interactions that embody an organization’s formal procedures, and the procedural fairness scale assesses the degree to which fair procedures are used in the organization [6]. All items are measured on a five-point Likert scale ranging from 1 (strongly disagree) to 5 (strongly agree). The score for each subscale is calculated by averaging the item scores. For this sample, Cronbach’s alpha coefficients were 0.94 for interactional fairness and 0.88 for procedural fairness.

To calculate the deviation of fairness perception, a categorical variable was generated that indicated if an individual’s fairness perception deviated from the group average. Specifically, individuals were categorized as having low deviation if their individual-level fairness perceptions were between one standard deviation and two standard deviations away from the group-level mean fairness perception. The individuals were categorized as having high deviation if their individual-level fairness perceptions were more than two standard deviations away from the group-level mean fairness perception [40]. This procedure is illustrated in the following equation:

$$ {\mathrm{deviation}}_i=\Big\{\begin{array}{l}0\ \left(\mathrm{no}\right),\mathrm{if}\ {\mu}_k-{\sigma}_k\le {x}_i\le {\mu}_k+{\sigma}_k\\ {}1\ \left(\mathrm{low}\right),\mathrm{if}\ {\mu}_k-2{\sigma}_k\le {x}_i<{\mu}_k-{\sigma}_k\ \mathrm{or}\ {\mu}_k+{\sigma}_k<{x}_i\le {\mu}_k+2{\sigma}_k\\ {}2\ \left(\mathrm{high}\right),\mathrm{if}\ {x}_i<{\mu}_k-2{\sigma}_k\ \mathrm{or}\ {x}_i>{\mu}_k+2{\sigma}_k\ \end{array}\operatorname{} $$

where *i* is the individual of interest and *k* is the reference group to which *i* belongs. The fairness perception of the individual *i* is expressed as *x*_*i*_, and *μ*_*k*_ and *σ*_*k*_ respectively indicate the mean and standard deviation of the fairness perception in group *k*.

As the current dataset did not contain information about work units, workers with the same demographic characteristics (i.e., company affiliation, age group, gender, and occupational class) were classified into the same reference groups. These variables were expected to be relevant for identifying reference groups of Japanese employees in relatively large companies because of a unique characteristic of the Japanese employment system, namely the “mass and simultaneous recruitment of new graduates” [41]. Under this tradition in the Japanese labor market, new college graduates are simultaneously hired at the same time of year. These new employees are treated as trainees and are provided with on-the-job training to enable them to acquire the skills, knowledge, and corporate culture necessary to become “company men” [42]. With this tradition, those who began working for the company in the same cohort would likely share an informal network throughout their entire employment period [25]. Drawing on this unique social structure in Japanese companies, we used company affiliation, age group, gender, and occupational class stratum to identify a reference group to which an employee compares her/his position.

The analysis excluded groups smaller than 20, assuming that group dynamics become more susceptible to the characteristics of individual workers rather than to the characteristics of a group when the group size is small. The robustness of this threshold was also confirmed using thresholds of 10 and 30, which yielded similar results.

#### Serious Psychological Distress

Serious psychological distress was measured using the Japanese version of the Kessler Psychological Distress (K6) scale, developed based on Kessler’s original K6 scale [43, 44]. The K6 scale consists of six items that ask respondents how often they have experienced symptoms of psychological distress during the past 30 days. The items are measured on a five-point Likert scale ranging from 0 (none of the time) to 4 (all of the time). Respondents with a total K6 score of 13 or more were categorized as having serious psychological distress and those with a score of 12 or less were categorized as not having serious psychological distress [45]. Cronbach’s alpha coefficient was 0.89 for the K6.

#### Covariates

Demographic characteristics and health-related behaviors were included as covariates. Demographic characteristics were gender (male or female) [12, 46], age group (18–29 years, 30–39 years, 40–49 years, 50–59 years, or 60 years or more) [12, 47], educational attainment (junior high school, high school or junior college, or college or graduate school) [12, 48], company [49], and type of work (day shift, shift work with night duty, shift work without night duty, or night shift) [50, 51].

Health-related behaviors were drinking (rarely, sometimes, or daily) [52, 53], smoking (never smoked, ex-smoker, or current smoker) [52, 54], and physical activity (none, light physical activity once or more per week, intense physical activity once or twice per week, or intense physical activity three times or more per week) [52, 55].

### Participants

**Fig. 1:**
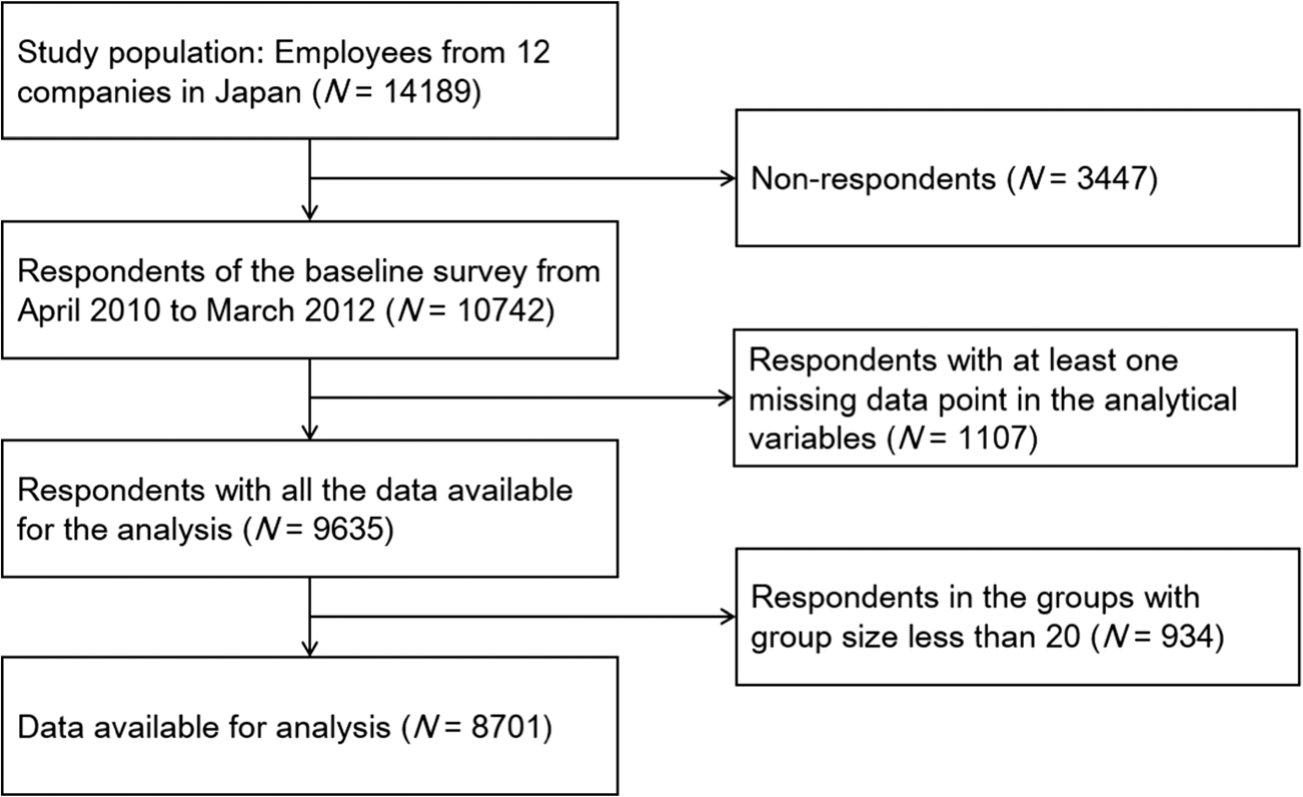
Flow diagram of the respondent selection process

The procedure for respondent selection is summarized in Fig. 1. Of the study population, 10,742 respondents completed the baseline survey from April 2010 to March 2012. After excluding those respondents with at least one missing response for the variables used in the analysis, 9635 respondents remained. After additionally excluding those respondents who belonged to groups of less than 20, the final number of respondents was 8701.

### Statistical Analysis

Multilevel logistic regression analyses were conducted to estimate the odds ratios (OR) and 95% confidence intervals (CI) of serious psychological distress. The intraclass correlation coefficient was 0.051, and the median odds ratio was 1.49. The likelihood-ratio test indicated that between-group variation is not negligible.

Five models were used in the analyses, and the analytical procedure was the same for both interactional fairness and procedural fairness. In the first step, the OR for deviation was calculated using a model without any adjustment for covariates (unadjusted model). In the second step, individual-level fairness perception was added to the model (model 1). In the third step, group-level mean fairness perception was added to the model (model 2). In the fourth step, demographic characteristics were added to the model (age group, gender, educational attainment, company, and type of work) (model 3). In the last step, health-related behaviors were also added to the model (drinking, smoking, and physical activity) (model 4).

All statistical analyses were performed using Stata software (Stata Corp., College Station, TX, USA), version 15.1.

## Results

Detailed descriptive statistics for individual-level variables (i.e., demographic characteristics, health-related behaviors, and other relevant variables) are shown in Table 1. The prevalence of serious psychological distress was 9.0%. The proportion of workers with deviating interactional fairness perceptions was 23.8% for low deviation and 4.6% for high deviation. The proportion of workers with deviating procedural fairness perceptions was 24.7% for low deviation and 5.2% for high deviation. Deviant group categories contained both positive and negative deviants, except for the high deviation of interactional fairness category, which was mainly composed of negative deviants. Group-level variables are shown in Table 2. The total number of groups was 101, and the group size ranged from 20 to 397 with a mean of 86.1.

**Table 1:** Descriptive statistics for individual-level variables

	Mean (SD)	*n* (%)
Demographic characteristics		
Gender		
Male		6958 (80.0)
Female		1743 (20.0)
Age	40.6 (10.3)	
60 years or more		151 (1.7)
50–59 years		1750 (20.1)
40–49 years		2961 (34.0)
30–39 years		2278 (26.2)
18–29 years		1561 (17.9)
Educational attainment		
College or graduate school		3824 (44.0)
High school or junior college		4690 (53.9)
Junior high school		187 (2.2)
Occupational class		
Manager		1535 (17.6)
Non-manual worker		3989 (45.9)
Manual worker		2235 (25.7)
Others		942 (10.8)
Type of work		
Day shift		7026 (80.8)
Shift work without night duty		1333 (15.3)
Shift work with night duty		205 (2.4)
Night shift		137 (1.6)
Health-related behaviors		
Drinking		
Rarely		3032 (34.9)
Sometimes		3053 (35.1)
Daily		2616 (30.1)
Smoking		
Never smoked		4990 (57.4)
Ex-smoker		1081 (12.4)
Current smoker		2630 (30.2)
Physical activity (PA)		
None		5391 (62.0)
Light PA once or more per week		1923 (22.1)
Intense PA once or twice per week		1100 (12.6)
Intense PA three times or more per week		287 (3.3)
Serious psychological distress (K6)	5.58 (4.74)	
No serious psychological distress		7915 (91.0)
Serious psychological distress		786 (9.0)
Fairness perception		
Interactional fairness	3.54 (0.83)	
Procedural fairness	3.21 (0.71)	
Deviation		
Interactional fairness		
No deviation		6226 (71.6)
Low deviation		2073 (23.8)
High deviation		402 (4.6)
Procedural fairness		
No deviation		6101 (70.1)
Low deviation		2148 (24.7)
High deviation		452 (5.2)

*SD* standard deviation

**Table 2:** Descriptive statistics for group-level variables

	Mean (SD)	*n*
Number of groups		101
Group size (20–397)	86.1 (74.2)	
Fairness perception (group mean)		
Interactional fairness	3.54 (0.24)	
Procedural fairness	3.21 (0.18)	

*SD* standard deviation

Table 3 presents the results for the analysis of the association between deviation of interactional fairness perceptions and serious psychological distress. For the model without any adjustment for covariates (unadjusted model), both low deviation (OR = 1.45, 95% CI; 1.22–1.72) and high deviation (OR = 4.68, 95% CI; 3.65–6.00) were significantly and positively associated with serious psychological distress.

**Table 3:** Association between deviation of interactional fairness perception and serious psychological distress

	Odds ratio (95% confidence interval)				
	Unadjusted model^a^	Model 1^b^	Model 2^c^	Model 3^d^	Model 4^e^
Fixed effect					
(Individual level)					
Deviation					
No deviation	1.00 (Reference)	1.00 (Reference)	1.00 (Reference)	1.00 (Reference)	1.00 (Reference)
Low deviation	1.45 (1.22–1.72)	1.24 (1.03–1.49)	1.24 (1.03–1.49)	1.23 (1.02–1.48)	1.24 (1.03–1.49)
High deviation	4.68 (3.65–6.00)	1.57 (1.12–2.19)	1.57 (1.12–2.19)	1.57 (1.12–2.19)	1.57 (1.12–2.19)
Fairness perception		0.57 (0.51–0.63)	0.57 (0.51–0.63)	0.56 (0.50–0.63)	0.56 (0.50–0.63)
(Group level)					
Mean fairness perception			1.01 (0.61–1.67)	0.54 (0.31–0.92)	0.54 (0.31–0.94)
Constant	0.07 (0.06–0.08)	0.07 (0.06–0.09)	0.07 (0.06–0.09)	0.24 (0.12–0.48)	0.28 (0.14–0.58)
	Variance (SE)				
Random effect					
Group-level intercept	0.200 (0.059)	0.192 (0.058)	0.192 (0.058)	0.030 (0.023)	0.030 (0.023)

*SE* standard error

^a^Without any adjustment

^b^Adjusted for individual-level fairness perception

^c^Additionally adjusted for group-level mean fairness perception

^d^Additionally adjusted for demographic characteristics (age group, educational attainment, company, and type of work)

^e^Additionally adjusted for health-related behaviors (drinking, smoking, and physical activity)

After adjustment for individual-level fairness perception (model 1), the OR for low deviation attenuated to 1.24 (95% CI; 1.03–1.49), and the OR for high deviation attenuated to 1.57 (95% CI; 1.12–2.19), but both remained significant. Individual-level fairness perception was significantly and negatively associated with serious psychological distress (OR = 0.57, 95% CI; 0.51–0.63).

Adjustment for group-level mean fairness perception had no effect on the ORs for low deviation, high deviation, and individual-level fairness perception (model 2). The OR for group-level mean fairness perception was not significant at this stage (OR = 1.01, 95% CI; 0.61–1.67).

Additional adjustment for demographic characteristics (model 3) did not remarkably affect the ORs for low deviation (OR = 1.23, 95% CI; 1.02–1.48) and high deviation (OR = 1.57, 95% CI; 1.12–2.19). The OR for individual-level fairness perception was not remarkably affected either (OR = 0.56, 95% CI; 0.50–0.63). The OR for group-level mean fairness perception turned to 0.54 (95% CI; 0.31–0.92), indicating a significant and negative association with serious psychological distress.

Further adjustment for health-related behaviors (model 4) did not make any remarkable difference in the coefficient estimations of fairness perceptions.

Table 4 shows the results for the analysis of the association between deviation of procedural fairness perception and serious psychological distress. For the model without any adjustment for covariates (unadjusted model), both low deviation (OR = 1.20, 95% CI; 1.01–1.43) and high deviation (OR = 3.06, 95% CI; 2.39–3.93) were significantly and positively associated with serious psychological distress.

**Table 4:** Association between deviation of procedural fairness perception and serious psychological distress

	Odds ratio (95% confidence interval)				
	Unadjusted model^a^	Model 1^b^	Model 2^c^	Model 3^d^	Model 4^e^
Fixed effect					
(Individual level)					
Deviation					
No deviation	1.00 (Reference)	1.00 (Reference)	1.00 (Reference)	1.00 (Reference)	1.00 (Reference)
Low deviation	1.20 (1.01–1.43)	1.03 (0.86–1.23)	1.02 (0.85–1.23)	1.01 (0.84–1.22)	1.02 (0.85–1.23)
High deviation	3.06 (2.39–3.93)	1.50 (1.10–2.04)	1.51 (1.11–2.06)	1.52 (1.12–2.08)	1.51 (1.11–2.06)
Fairness perception		0.50 (0.44–0.57)	0.50 (0.45–0.57)	0.50 (0.44–0.57)	0.50 (0.44–0.57)
(Group level)					
Mean fairness perception			0.43 (0.22–0.83)	0.51 (0.24–1.06)	0.51 (0.24–1.07)
Constant	0.08 (0.07–0.09)	0.08 (0.07–0.09)	0.08 (0.07–0.09)	0.24 (0.12–0.48)	0.28 (0.14–0.57)
	Variance (SE)				
Random effect					
Group-level intercept	0.180 (0.056)	0.193 (0.059)	0.185 (0.056)	0.032 (0.023)	0.033 (0.023)

*SE* standard error

^a^Without any adjustment

^b^Adjusted for individual-level fairness perception

^c^Additionally adjusted for group-level mean fairness perception

^d^Additionally adjusted for demographic characteristics (age group, educational attainment, company, and type of work)

^e^Additionally adjusted for health-related behaviors (drinking, smoking, and physical activity)

After adjustment for individual-level fairness perception (model 1), the OR for low deviation attenuated to 1.03 (95% CI; 0.86–1.23), indicating only a weak association with serious psychological distress, whereas the OR for high deviation remained significant (OR = 1.50, 95% CI; 1.10–2.04). Individual-level fairness perception was significantly and negatively associated with serious psychological distress (OR = 0.50, 95% CI; 0.44–0.57).

Adjustment for group-level mean fairness perception (model 2) slightly attenuated the OR for low deviation (OR = 1.02, 95% CI; 0.85–1.23), while there was a small increase in the OR for high deviation (OR = 1.51, 95% CI; 1.11–2.06). The OR for individual-level fairness perception was not remarkably affected. The group-level mean fairness perception was significantly and negatively associated with serious psychological distress (OR = 0.43, 95% CI; 0.22–0.83).

Additional adjustment for demographic characteristics did not have remarkable effect on the OR for low deviation, high deviation, and individual-level fairness perception (model 3). The OR for group-level mean fairness perception became non-significant (OR = 0.51, 95% CI; 0.24–1.06), but still indicated a weak and negative association with serious psychological distress.

Further adjustment for health-related behaviors (model 4) did not remarkably affect the ORs for low deviation, high deviation, individual-level fairness perception, and group-level mean fairness perception.

## Discussion

The present study examined the cross-sectional association between individual deviation of fairness perception from the group average and serious psychological distress. To summarize the key findings, both low deviation and high deviation of interactional fairness perception were significantly and positively associated with serious psychological distress, independently of individual-level fairness perception, group-level mean fairness perception, demographic characteristics, and health-related behaviors. Only high deviation of procedural fairness perception was significantly and positively associated with serious psychological distress.

### Interpretations

The results confirmed that perception of fairness, both at individual and group levels, had a negative association with serious psychological distress. This result is consistent with findings from previous studies. For interactional fairness, Inoue et al. found that individual-level fairness perception was negatively associated with psychological distress [7], and Moliner et al. found that group-level mean fairness perception was negatively associated with group-level burnout [17]. For procedural fairness, Kivimäki et al. found a negative association between fairness perception and psychiatric disorder at both individual level and work unit levels [15]. In addition, Grynderup et al. found that group-level mean fairness perception—for both interactional fairness and procedural fairness—was negatively associated with interview-diagnosed onset of depression after 2 years [56]. The current study added a new insight that deviation of both interactional and procedural fairness perceptions from the reference group norms was significantly and positively associated with serious psychological distress.

The association between deviation of procedural fairness perception and serious psychological distress was only observed for high deviation, and the effect was smaller than that of interactional fairness; this result warrants further explanation. Previous studies have suggested that compared with moral deviations that are impersonal, personal moral deviations are more harmful, because they directly affect related individuals [19, 57]. Interactional fairness mainly reflects workers’ evaluations of their supervisors’ interpersonal treatment in the worksite. In contrast, procedural fairness is related to decision-making procedures and thus reflects more structural aspects of the worksite. These conceptual differences suggest that group cohesiveness and integrity are more likely to be threatened by the presence of workers with deviating interpersonal fairness perceptions, whereas deviating procedural fairness perceptions may have only a small effect.

### Implications

Previous studies have recommended population-based interventions for worksite fairness, such as supervisory and managerial training, to promote fairer interpersonal treatment [18, 35]. The results of the current study also support improvements in the group average of worksite fairness for protecting workers from serious psychological distress. However, this study further indicated that the protective effect would be reduced if group improvement left some workers behind in exclusion and isolation.

Greater attention has recently been paid to the inclusion of diverse workers in the worksite [58]. Shore et al. defined inclusion as the degree to which workers’ belongingness and uniqueness are assured, so that workers feel esteemed by members of their work groups [59]. In contrast to the detrimental effect of exclusion [34], inclusion has positive effects on workers’ health [60]. Therefore, in addition to traditionally recommended interventions for worksite fairness, it would also be important to promote inclusive practices and a worksite climate in which diverse perceptions about fairness are allowed and encouraged.

### Limitations

The current study has several limitations. First, this study created groups using demographic and occupational class information, because the current dataset did not contain information about work units. However, these information are expected to be relevant for identifying reference groups of workers, given the unique characteristic of the Japanese employment system discussed earlier [41]. In contrast to work units, which represent formal groups within a company [23], these reference groups are informal groups that may have distinct sets of norms shared by their members [61]. Therefore, it might be possible that deviation in work units and deviation in reference groups independently affect the health of workers; this possibility warrants further investigation in the future research.

Second, the results may have been subject to common method bias owing to the measurement method used. In this study, both fairness perception and serious psychological distress were measured using self-reports, which can have a confounding effect on associations between variables [62]. Although some studies have reported minimal influence of common method bias [63], future research may benefit from using objective measures, such as objective diagnosis of psychiatric disorders and other physiological measures.

Third, cross-sectional design may be weaker in causal inferences compared to prospective design [12, 17]. However, when the time interval between exposure and outcome is short, and when the exposure changes in a short period of time, careful selection of time lag between exposure and outcome is required in a prospective design [62]. As the J-HOPE study has 1-year time lags between waves, we could have alternatively employed a prospective design with 1-year observation period. However, psychological distress in response to job stressors often occurs within a year [64], and the deviation status can vary over time due to external pressures to comply with the group norm [29]. In fact, we confirmed that deviation status varied over different waves (data not shown). Consequently, we specifically chose a cross-sectional design, rather than prospective design with 1-year time lag, to sensitively capture the association between deviation of fairness perception and serious psychological distress. Nevertheless, future study can use a different measurement for deviation, e.g., external evaluation of deviation, to better account for common method bias, which may not be fully controlled in a cross-sectional study.

Fourth, caution is necessary in generalizing the findings of the current study to other settings or to all Japanese workers. The study population comprised full-time workers from 12 companies in Japan, which represent only some of the industries in the country. Therefore, further investigation is necessary to clarify if the present findings can be generalized to other settings and other types of work conditions.

Fifth, although positive deviants and negative deviants might be treated differently at worksites [65], the present study was unable to explore this possibility because of data limitations. In this study, differentiating positive deviants from negative deviants would result in multicollinearity in the model; participants with high individual-level fairness perceptions were more likely to be classified as positive deviants and vice versa. This problem arises because the only information available for classification in the current dataset is the participant’s own perceptions, instead of how others evaluate them. Therefore, future research could measure how participants evaluate deviating members. This measure would enable the examination of whether positive deviants and negative deviants are treated differently at worksites, as well as the effects of this treatment on their health.

### Conclusion

In conclusion, this study demonstrated an association between deviation of interactional and procedural fairness perception and serious psychological distress that were independent of individual-level fairness perception or group-level mean fairness perception. The results suggest that deviation of interactional and procedural fairness perception could be the alternative pathways by which interactional fairness and procedural fairness affect worker health. The findings also suggest that, in addition to improvements in the group average of worksite fairness, inclusive treatment for individuals with divergent perceptions within a group is needed to achieve social inclusion of minority voices for worksite fairness.
